# Activating transcription factor 3 is crucial for antitumor activity and to strengthen the antiviral properties of Onconase

**DOI:** 10.18632/oncotarget.14302

**Published:** 2016-12-27

**Authors:** Anna Vert, Jessica Castro, Marc Ribó, Antoni Benito, Maria Vilanova

**Affiliations:** ^1^ Laboratori d’Enginyeria de Proteïnes, Departament de Biologia, Facultat de Ciències, Universitat de Girona, Campus de Montilivi, 17003, Girona, Spain; ^2^ Institut d’Investigació Biomèdica de Girona Josep Trueta, (IdIBGi), Girona, Spain

**Keywords:** antitumor and antiviral drug, Onconase, activating transcription factor 3, microarray profiling, apoptosis

## Abstract

Onconase is a ribonuclease that presents both antitumor and antiviral properties linked to its ribonucleolytic activity and represents a new class of RNA-damaging drugs. It has reached clinical trials for the treatment of several cancers and human papilloma virus warts. Onconase targets different RNAs in the cell cytosol but Onconase-treated cells present features that are different from a simple arrest of protein synthesis. We have used microarray-derived transcriptional profiling to identify Onconase-regulated genes in two ovarian cancer cell lines (NCI/ADR-RES and OVCAR-8). RT-qPCR analyses have confirmed the microarray findings. We have identified a network of up-regulated genes implicated in different signaling pathways that may explain the cytotoxic effects exerted by Onconase. Among these genes, activating transcription factor 3 (ATF3) plays a central role in the key events triggered by Onconase in treated cancer cells that finally lead to apoptosis. This mechanism, mediated by ATF3, is cell-type independent. Up-regulation of ATF3 may also explain the antiviral properties of this ribonuclease because this factor is involved in halting viral genome replication, keeping virus latency or preventing viral oncogenesis. Finally, Onconase-regulated genes are different from those affected by nuclear-directed ribonucleases.

## INTRODUCTION

Onconase (ONC), a protein of amphibian origin, is a member of the vertebrate secreted ribonucleases (RNases) family that presents antitumor and antiviral activities. Its structure endows it with unusual high conformation stability [1] and with the ability to evade the cytoplasmic ribonuclease inhibitor (RI), a 50kDa protein that tightly binds to some RNases and inhibits their activity [2]. Both its conformational stability and its ability to evade the RI provide ONC with significant intracellular survival that is critical to its biological actions.

Regarding the antitumor activity, it has been shown that ONC exhibits selective cytostatic and cytotoxic activities [3] against many human tumor models (for a review see [4, 5]). In addition, ONC presents synergy with a significant number of anti-tumor drugs, such as tamoxifen, lovastatin, cisplatin, and vincristine, among others (for a review see [6]). More interestingly, it has entered clinical trials for the treatment of different types of cancer and has reached phase II/III for the treatment of malignant mesothelioma [7–9].

The ONC-induced apoptotic effects linked to its cell internalization mechanism are still not well understood. It is believed that the normal route for ONC to reach the cytosol is through an endosomal compartment, but controversy exists about the existence of a cellular receptor [6]. Once in the cytosol, ONC degrades RNA, thereby preventing protein synthesis and inducing cell cycle arrest and apoptosis. However, ONC-induced apoptosis presents features different from those of indiscriminate protein synthesis arrest (for a review see [6]). In this regard, it has been demonstrated that, besides rRNA, the initially proposed ONC target [10], mRNAs [11, 12], tRNAs [13], and miRNAs or their precursors [14–16] are also ONC targets. Accordingly, ONC up- and down-regulates genes that code for proteins involved in cell cycle progression or transcription factors involved in cell survival [11, 12, 17]. In addition, ONC may trigger cell death in several ways, depending on the cell type. It may induce caspase-dependent apoptosis linked to the activation of the stress-activated protein kinase c-Jun NH2 [18] or intrinsic apoptosis together with the activation of Ser-proteases [19, 20]. It has been demonstrated that intrinsic apoptosis depends on Apaf-1 reversing the inhibitory effect of tRNAs on the association of cytochrome c with Apaf-1 [21]. In addition, ONC may induce cell death by autophagy [22, 23].

The antiviral activity of ONC is of particular interest due to ONC's ability to degrade not only RNA but dsRNA [24]. Antiviral drugs that destroy viral genomes not only suppress virus production but are likely to abolish latent virus infection. It has been described that ONC exhibits efficacy in suppressing the replication of HIV-1, leaving non-infected cells unharmed [25]. Recently, several patents have been obtained by Tamir Biotechnology, Inc. (San Diego, CA) (http://tamirbio.com/) to use wild type ONC and variants as antiviral agents. The most success has been obtained with human papilloma virus (HPV) [26], although other viral infections, such as those of the Herpesviridae family, are also being treated [27]. ONC is now in clinical trial to study its safety and efficacy by topical administration to treat genital warts (clinical trial identifier: NCT02535104 at https://clinicaltrials.gov/). What is more, ONC is being considered alone or as adjuvant therapy in an effort to fight Ebola, orthomyxoviruses (influenza), Middle East Respiratory Syndrome (MERS-CoV), the Hepatitis C virus (HCV), the cytomegalovirus (CMV), the respiratory syncytial virus (RVS), and rhabodviruses (rabies) (http://tamirbio.com/).

Taking into account the large number of diseases with high social impact that are or can be treated with ONC, we have used microarray technology to identify significant altered gene expression in the treatment of two ovarian carcinoma cell lines, NCI/ADR-RES and its parental OVCAR-8, with this enzyme. The present work is the second to use microarray-derived transcriptional profiling to identify ONC-regulated genes in order to understand the cytotoxic mechanism of this RNase. In a previous work, Altomare et al. [12] investigated the effect of ONC on different human malignant mesothelioma (hMM) cell lines and showed that this RNase alters the expression of genes that control the cell cycle and apoptosis.

Our results show that the ONC up-regulated activating transcription factor 3 (ATF3) is a cell-type independent key factor of ONC antitumor activity. ATF3 activates other transcription factors that control different signaling pathways. This explains the effects observed in the cell cycle and the induction of apoptosis in cells treated with ONC. Interestingly, it has been described that ATF3 and some of its regulated genes help to preclude viral genome replication, to maintain virus latency, or to prevent viral oncogenesis. Finally, our results also show that the pleiotropic effects caused by ONC, derived from its ribonucleolytic activity in the cytosol, are completely different from those promoted by nuclear-directed RNases (ND-RNases) [28].

## RESULTS

ONC is a paradigmatic antitumor RNase whose cytotoxic mechanism has been studied by different means [6]. Nevertheless, microarray profiling to broadly analyze the genes affected by this drug has only been done in hMM cell lines [12]. We have used the same approach to study the effects of ONC in two ovarian carcinoma cell lines, NCI/ADR-RES and its parental OVCAR-8. In addition, our results allow us to compare the effects on gene expression promoted by two different RNases in cancer cells: ONC, an RNase that exerts its action in the cytosol, and PE5, an ND-RNase [29].

### Cell proliferation and RNase sensitivity

Since ONC has ribonucleolytic activity, an unbiased approach to examine gene expression by microarray technology requires searching for optimal cell treatment conditions. An RNA population representative of the actual drug effect must be obtained, not one that results from an extensive RNA degradation caused by the apoptosis process [30]. Therefore, we investigated the cytotoxic effect of ONC on NCI/ADR-RES and OVCAR-8 cells at different ONC concentrations and incubation times, as we had previously done with PE5 [28]. A dose-dependent cytotoxic effect was apparent after 24 h of incubation with ONC and increased with time (Supplementary Figure 1 Supplementary Data). As expected [31], the cytotoxic effect of ONC was greater than that of PE5 [28], and the MDR cell line, NCI/ADR-RES, was less sensitive to both RNases than its parental cell line OVCAR-8.

Next, we analyzed the RNA degradation caused by different doses of ONC. We incubated NCI/ADR-RES and OVCAR-8 cells with ONC concentrations that induced a decrease in cell proliferation of 5%, 10%, and 15% (IC_5_, IC_10_, and IC_15_). After 36 h of incubation, the RNA degradation of treated and untreated cells was quantified using a bioanalyzer. The RNA integrity number (RIN) values obtained for all samples were higher than 9.3. For a good comparison of the effects of ONC and PE5 on the NCI/ADR-RES cell line, we decided to carry out the microarray experiments at that concentration of ONC that triggered the same decrease in cell viability (10%) observed with PE5 [28] after 36 h of treatment (i.e. 0.5 μM). Likewise, we chose an ONC concentration for the OVCAR-8 cell line of 0.06 μM, which also produced a 10% decrease in cell proliferation.

### Gene expression changes in ONC-treated cells

ONC-treated and untreated NCI/ADR-RES cells revealed 56 differentially expressed genes out of 35,377 present in the microarray (1.6 × 10^-3^ %). Among them, 89% were up-regulated (the increase from untreated cells ranged from 2- to 6.5-fold), whereas 11% were down-regulated (the decrease from untreated cells ranged from 2- to 2.5-fold). Table 1 shows the top 20 ONC up-regulated genes as well as all the ONC down-regulated genes. The results indicate that the primary effect of ONC in NCI/ADR-RES cells is the activation of gene expression. ONC up-regulated genes are involved in transcription regulation (ATF3, CREB5, EGR1), cell cycle and apoptosis (GADD45A, PPP1R15A), immune response (IL6, IL33, IL1RL1, IL23A), and stress response (TXNIP), among other processes. Some of the up-regulated genes act as tumor suppressors (TXNIP, EGR1, and PPP1R15A). ONC down-regulated genes are also associated with transcription regulation (RDBP, XBP1), amino acid metabolism (PHGDH, ASNS), and protein folding (HSPA1A, HSPA8).

**Table 1:** ONC differentially expressed genes in NCI/ADR-RES cell line: top 20 ONC up-regulated genes and the 6 down-regulated genes

Probe ID	Gene Symbol	Gene Name	Fold Change	Main Functions
A_23_P97700	TXNIP	Thioredoxin interacting protein	6.5	Transcription regulationCell cycleCell proliferationStress responseApoptosis
A_23_P34915	ATF3	Activating transcription factor 3	6.5	Transcription regulation
A_23_P157117	CREB5	cAMP responsive element binding protein 5	6.2	Transcription regulation
A_23_P167983	HIST1H2AC	Histone cluster 1, H2ac	4.0	Nucleosome assembly
A_23_P339818	ARRDC4	Arrestin domain containing 4	3.9	Signal transduction
A_23_P328740	NEURL3	Neuralized homolog 3 (Drosophila) pseudogene	3.6	Transcription regulation
A_23_P23221	GADD45A	Growth arrest and DNA-damage-inducible, alpha	3.2	Cell cycleDNA repairApoptosis
A_23_P71037	IL6	Interleukin 6 (interferon, beta 2)	3.2	Immune responseCell differentiationCell proliferationApoptosisTranscription regulation
A_23_P259071	AREG	Amphiregulin	3.2	Cell proliferation
A_23_P92909	SPINK6	Serine peptidase inhibitor, Kazal type 6	3.0	Epidermis development
A_23_P214080	EGR1	Early growth response 1	3.0	Transcription regulation
A_23_P90172	PPP1R15A	Protein phosphatase 1, regulatory subunit 15A	2.7	ApoptosisStress responseTranslation regulationCell cycle
A_32_P18440	ARID5B	AT rich interactive domain 5B (MRF1-like)	2.5	Transcription regulation
A_32_P141238	ANO2	Anoctamin 2	2.5	Ion transport
A_23_P139500	BHLHE41	Basic helix-loop-helix family, member e41	2.5	Transcription regulation
A_23_P321501	DHRS2	Dehydrogenase/reductase (SDR family) member 2	2.5	Stress response
A_23_P320739	MEF2C	Myocyte enhancer factor 2C	2.5	Transcription regulationCell differentiationImmune response
A_33_P3243887	IL11	Interleukin 11	2.4	Cell differentiationCell proliferationTranscription regulation
A_24_P932736	HMBOX1	Homeobox containing 1	2.4	Transcription regulation
A_23_P51126	IL1RL1	Interleukin 1 receptor-like 1	2.4	Immune response
A_33_P3390758	HSPA8	Heat shock 70kDa protein 8	-2.0	Protein foldingUnfolded protein response
A_23_P120845	XBP1	X-box binding protein 1	-2.2	Transcription regulationUnfolded protein response
A_23_P111132	HSPA1A	Heat shock 70kDa protein 1A	-2.3	Protein foldingUnfolded protein response
A_23_P145694	ASNS	Asparagine synthetase (glutamine-hydrolyzing)	-2.3	Amino acid metabolismUnfolded protein response
A_23_P85783	PHGDH	Phosphoglycerate dehydrogenase	-2.5	Amino acid metabolism
A_23_P122545	RDBP	RD RNA binding protein	-2.5	Transcription regulation

Gene information was taken from the UniProt database (European Bioinformatics, UK, Swiss Institute of Bioinformatics, Switzerland, Protein Information Resource, USA) (http://www.uniprot.org) and from the Entrez Gene database (National Center for Biotechnology Information, USA) (http://www.ncbi.nlm.nih.gov/gene).

ONC-treated and untreated OVCAR-8 cells revealed seven differentially expressed genes out of 35,377 present in the microarray (1.8 × 10^-4^ %). All of them were up-regulated (the increase from untreated cells ranged from 2- to 4-fold) (Table 2), indicating that the primary effect of ONC is also gene expression activation. Again, some of the up-regulated genes are related to transcription regulation (ATF3, NEURL3, ZNF750), while others are involved in stress response (TXNIP). Six out of the seven ONC differentially expressed genes in the OVCAR-8 cell line were also differentially expressed in the NCI/ADR-RES cell line (TXNIP, ATF3, HIST1H2AC, NEURL3, DHRS2, and NPPB).

**Table 2:** ONC differentially expressed genes in OVCAR-8 cell line

Probe ID	Gene Symbol	Gene Name	Fold Change	Main Functions
A_23_P34915	ATF3	Activating transcription factor 3	4.0	Transcription regulation
A_23_P97700	TXNIP	Thioredoxin interacting protein	3.2	Transcription regulationCell cycleCell proliferationStress responseApoptosis
A_23_P328740	NEURL3	Neuralized homolog 3 (Drosophila) pseudogene	2.9	Transcription regulation
A_33_P3423420	ZNF750	Zinc finger protein 750	2.3	Transcription regulation
A_23_P167983	HIST1H2AC	Histone cluster 1, H2ac	2.3	Nucleosome assembly
A_23_P321501	DHRS2	Dehydrogenase/reductase (SDR family) member 2	2.2	Stress response
A_23_P62752	NPPB	Natriuretic peptide B	2.1	Cardiovascular homeostasis

Gene information was taken from the UniProt database (European Bioinformatics, UK, Swiss Institute of Bioinformatics, Switzerland, Protein Information Resource, USA) (http://www.uniprot.org) and from the Entrez Gene database (National Center for Biotechnology Information, USA) (http://www.ncbi.nlm.nih.gov/gene).

Among the ONC and PE5 differentially expressed genes in the NCI/ADR-RES cell line, we identified only 10 that were common for both RNase treatments (i.e. 1.5% and 18% of the PE5 [28] and ONC differentially expressed genes, respectively). Eight of them were up-regulated in RNase-treated cells respective to untreated cells, whereas the other two were down-regulated. Common up-regulated genes are related to transcription regulation (HMBOX1, LINC00340, NEXN-AS1), immune response (CD86, IL6, IFIT2) and the last two are potential tumor suppressors (LRRC2 and SPINK6 [32, 33]). Common down-regulated genes participate in amino acid metabolism (PHGDH, ASNS).

### Quantitative real-time PCR analysis of gene expression

**Figure 1:**
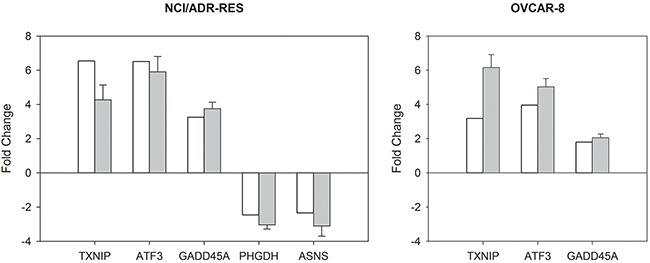
Quantitative comparison of gene expression changes in NCI/ADR-RES and OVCAR-8 cell lines The histograms show the fold change values of selected genes (gene intensities of treated relative to control samples) obtained by microarrays (white) and RT-qPCR (grey) in NCI/ADR-RES and OVCAR-8 cell lines. RT-qPCR data are presented as mean ± SD. Genes: Thioredoxin interacting protein (TXNIP), activating transcription factor 3 (ATF3), growth arrest and DNA-damage-inducible, alpha (GADD45A), phosphoglycerate dehydrogenase (PHGDH), and asparagine synthetase (glutamine-hydrolyzing) (ASNS).

mRNA expression of three ONC up-regulated (TXNIP, ATF3, and GADD45A) and two down-regulated (PHGDH, ASNS) genes, representatives of the processes affected by this RNase, was analyzed by RT-qPCR. The results are presented in Supplementary Figure 2 of the Supplementary Data. Fold changes obtained with RT-qPCR, calculated as the ratio between relative transcript abundance (RTA) values obtained for ONC-treated cells and RTA values obtained for untreated cells, were similar to those found in the microarray analysis (Figure 1), confirming that the microarray experiments are fully valid.

### Gene ontology analysis and KEGG pathway annotation

To better understand the functional relevance of ONC-regulated genes in NCI/ADR-RES cells, we performed a gene ontology analysis. To the same end, ONC differentially expressed genes were mapped to the KEGG database to find over-represented known metabolic and regulatory pathways. These analyses were not done for the OVCAR-8 cell line because ONC-treatment gave only 10 differentially expressed genes. The NCI/ADR-RES differentially expressed genes were used to find over-represented gene ontology terms in the three broad ontology categories: “molecular function”, which captures knowledge about the functional activity of gene products, the larger “biological process”, as part of which these specific functions collectively act, and “cellular component”, where all this occurs. Gene ontology analysis shows that ONC differentially expressed genes (Table 3) are involved in a diversity of cellular events. The same was found for PE5, showing that these RNA-damaging drugs have pleiotropic effects [28].

**Table 3:** Gene ontology analysis and KEGG pathway annotation of ONC differentially expressed genes in NCI/ADR-RES cell line

Analysis	Term	Gene count ^a^	P-value
Gene Ontology - Biological Process	Cell proliferation	15	2.45E-05
	Response to stress	21	3.18E-05
	Signal transduction	24	2.03E-04
	Cell differentiation	18	4.34E-04
	Apoptotic process	13	4.81E-04
	Developmental process	24	7.50E-04
	Immune response	10	9.98E-04
	Transcription, DNA-dependent	18	3.44E-03
	Phosphorylation	10	3.95E-03
	Cell-cell signaling	9	4.46E-03
	Chromatin assembly	3	5.46E-03
	Protein metabolic process	20	5.80E-03
	Intracellular protein kinase cascade	7	1.45E-02
	Gene expression	20	2.20E-02
	Angiogenesis	4	2.24E-02
	Growth	6	2.66E-02
	RNA metabolic process	18	2.84E-02
	Cell migration	6	3.94E-02
	DNA metabolic process	6	4.52E-02
Gene Ontology - Molecular Function	Cytokine activity	4	3.66E-03
	Nucleic acid binding transcription factor activity	8	1.10E-02
	Growth factor activity	3	1.38E-02
	Hormone activity	2	4.48E-02
Gene Ontology - Cellular Component	Extracellular region	15	6.05E-04
	Cell surface	6	2.35E-03
	Nucleus	24	2.30E-02
KEGG pathway annotation	Prion diseases	3	4.59E-04
	Rheumatoid arthritis	4	6.22E-04
	MAPK signaling pathway	6	9.09E-04
	Systemic lupus erythematosus	4	2.77E-03
	Protein processing in endoplasmic reticulum	4	5.55E-03
	Graft-versus-host disease	2	1.39E-02
	Intestinal immune network for IgA production	2	1.88E-02
	JAK-STAT signaling pathway	3	3.01E-02
	Antigen processing and presentation	2	4.40E-02

^a^ Number of differentially expressed genes that belong to these terms.

The most ONC-affected biological processes were cell proliferation, response to stress, signal transduction, cell differentiation, and apoptosis. The individual function of ONC-regulated genes favored cytokine activity, nucleic acid binding transcription factor activity, and growth factor activity, and were mainly associated with the extracellular region, the cell surface, and the nucleus.

Analysis of the over-represented pathways collected in the KEGG database showed that ONC-affected pathways (Table 3) were involved in growth and development processes, like JAK-STAT or the MAPK signaling pathway, which affect basic cellular functions such as cell growth, proliferation, differentiation, and death, as well as stress response. Some pathways related to infection or immunological diseases were also affected.

## DISCUSSION

### ONC cytotoxic mechanism

**Figure 2:**
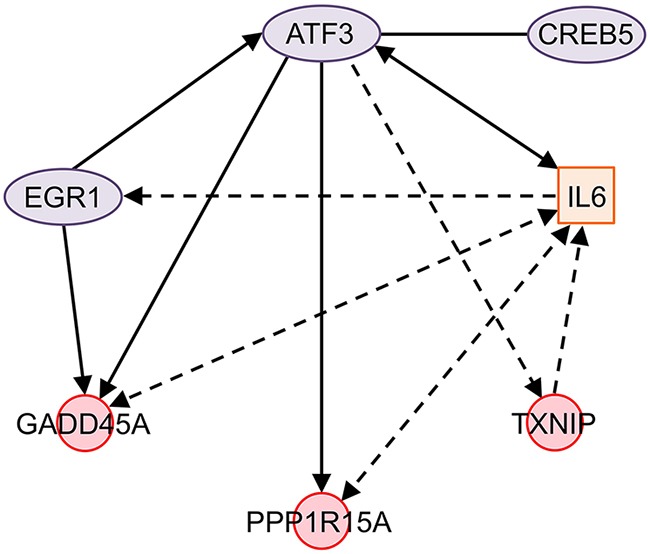
Network of ONC-regulated genes in NCI/ADR-RES cells ATF3 directly promotes the expression of GADD45A, PPP1R15A, and IL6. It indirectly increases the expression of TXNIP and EGR1 and binds to CREB5. ATF3 and GADD45A expression is increased by EGR1. IL6 affects the expression of ATF3 and indirectly that of EGR1. At the same time, IL6 indirectly affects and is affected by PPP1R15A and GADD45A, and it is also indirectly affected by TXNIP. Solid line: interaction between two genes or the proteins coded by them; solid arrows: direct effect on gene expression; dotted arrows: indirect effect on gene expression; solid double arrows: reciprocal direct effect on gene expression; dotted double arrows: reciprocal indirect effect on gene expression, no physical contact between them or the proteins coded by them is required; ovals: transcription regulators; squares: cytokines; circles: other functions. Figure designed using the Ingenuity Pathway Analysis (IPA) package available online at http://www.ingenuity.com/products/ipa Genes: ATF3, activation transcription factor 3; CREB5, cAMP responsive element binding protein 5; EGR1, early growth response 1; GADD45A, growth arrest and DNA damage-inducible, alpha; IL6, interleukin 6; PPP1R15A, protein phosphatase 1, regulatory subunit 15A; TXNIP, thioredoxin-interacting protein.

Gene ontology analysis and KEGG pathway annotation of ONC-differentially expressed genes in the NCI/ADR-RES cell line show interesting terms that allow us to understand how ONC induces cell death. These terms are cell proliferation, response to stress, and apoptotic processes (Table 3). In these fields we can find 7 of the 20 most ONC up-regulated genes in NCI/ADR-RES cells (Table 1) that correspond to functionally related transcription regulators: early growth response 1 (EGR1), activating transcription factor 3 (ATF3), growth arrest and DNA-damage-inducible, alpha (GADD45A), thioredoxin interacting protein (TXNIP), cAMP responsive element binding protein 5 (CREB5), and protein phosphatase 1, regulatory subunit 15A (PPP1R15A). Their relationship is depicted in Figure 2.

ATF3 is a member of the ATF/CREB family of transcription factors that transduces signals from various receptors to activate or repress gene expression. Depending on the cell type and context it plays different roles in cancer development [34]. Nevertheless, its ectopic expression induces apoptosis in a variety of cancer cells [35, 36], enhances the ability of different drugs, such as camptothecin, etoposide, curcumin, cisplatin and lovastatin, to induce apoptosis [37–40], and even inhibits the invasion and metastasis of cancer cells [41–43]. ATF3 was first described as associated with ONC activity by Altomare et al. [12]. Our results seem to indicate that it plays a central role in the ONC action because it directly up-regulates the expression of GADD45A [44] and PPP1R15A [45]. Additionally, it can indirectly increase TXNIP expression, since it promotes up-regulation of the Krüppel-like factor 6 (KLF6) [44], a transcription factor whose overexpression in turn increases that of TXNIP [46]. KLF6 is also up-regulated by ONC, although the fold change is lower than 2. Moreover, ATF3 can bind to CREB5 to form a heterodimer [47]. Although the physiological significance of this dimer is not clear, the general view is that its formation can alter DNA binding specificity and transcriptional activity, thus expanding the ability of this transcription factor to regulate gene expression [34]. Finally, it is described that the expression of ATF3 [48] and GADD45A [49] is increased by EGR1 (Figure 2). EGR1 is expressed at low levels in several types of cancer [50–52], acting as a tumor suppressor and inducing apoptosis of cancer cells in a p53-independent manner [53]. It is interesting to note that ONC exerts its cytotoxicity independently of the p53 phenotype [54].

GADD45A and PPP1R15A belong to the family of growth arrest and DNA damage (GADD) proteins, which are crucial to cellular stress responses [55]. It is known that GADD45A inhibits cell growth through the G_2_/M cell cycle arrest [56, 57] while PPP1R15A plays a vital role in promoting cell death following proteasome inhibition via enhancing protein synthesis to activate mechanisms associated with dead, including ER stress, ROS production, and autophagy formation [58]. In addition, down-regulation of GADD45A promoted by the NF-κB transcription factor is essential for cancer cell survival [59]. In this regard, it is worth mentioning that ONC mediates some of its effects by reducing cellular levels of NF-κB. In Jurkat cells, ONC induces an arrest of proliferation, accompanied by an altered subcellular distribution and reduced expression of NF-κB [17]. In addition, in hMM cells ONC inhibits NF-κB nuclear translocation induced by TNF-α [60] and produces a down-regulation of the NFKB1 gene, which encodes the NF-κB subunit p50, mediated by the increase in hsa-miR-17* and the decrease in hsa-miR-30c levels [61]. It can be expected that, in these cell lines, the inactivation of NF-κB would also lead to the up-regulation of GADD45A.

TXNIP, a protein with multiple functions, plays an important role in redox homeostasis [62]. Extensive evidence shows that TXNIP acts as a tumor suppressor [63–69]. ONC induces the expression of two ATF3-controlled genes whose encoded proteins promote cell cycle arrest at different checkpoints (G2/M in the case of GADD45A and G0/G1 in the case of TXNIP [64, 70]). This effect would be in accordance with previous results showing that ONC arrests cell growth of Jurkat and NCI/ADR-RES cells without altering the percentage of cells in the different cell cycle phases [17, 31].

**Figure 3:**
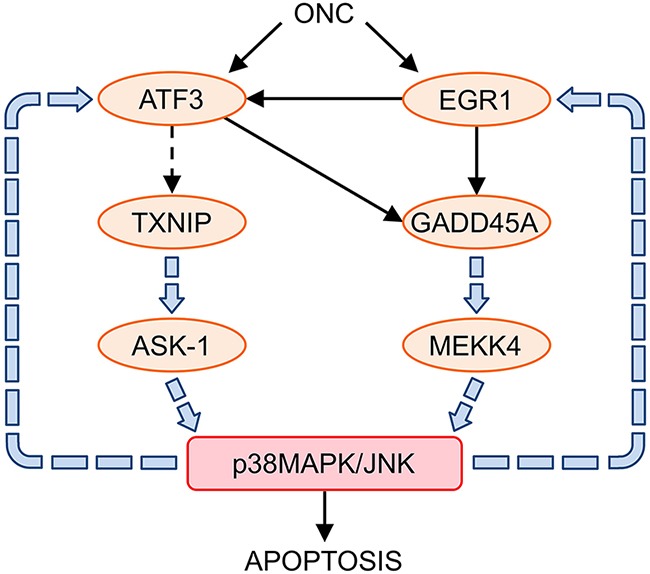
Relationship between ONC up-regulated genes and the p38MAPK/JNK signaling pathways ONC up-regulated and interconnected ATF3, EGR1, TXNIP, and GADD45A genes, activate p38MAPK/JNK signaling pathways through ASK-1 and MEKK4, which leads to cell apoptosis. The activation of p38MAPK/JNK reinforces the expression of the ONC up-regulated genes: ATF3 and EGR1.

ONC-regulated genes significantly affect the MAPK signaling pathway (Table 3). In this regard, it is noteworthy that TXNIP, through ASK1 activation [71], and GADD45A, through its interaction with mitogen-activated protein kinase kinase kinase 4 (MEKK4), are involved in the apoptosis induced by the p38 MAPK/JNK signaling pathway [72]. At the same time, the activation of p38 MAPK/JNK induces the expression of EGR1 [73, 74] and ATF3 [75], reinforcing the initial action of ONC thorough the up-regulation of these two genes (Figure 3). Accordingly, ONC is a potent activator of the stress-activated protein kinases JNK1, JNK2 and p38 MAPK in primary and immortalized fibroblasts [18].

In this work we show that, in addition to the MAPK pathway, ONC affects the Jak-STAT signaling pathway by altering the expression of different genes. Interestingly, it has been previously described that the cytotoxic effect of ONC on hMM cells is also mediated by the Jak-STAT pathway [12]. Regarding the Jak-STAT signaling, ONC treated NCI/ADR-RES cells show an increased expression of interleukin 6 (IL6) (Table 1 and Figure 2), previously observed in hMM-treated cells [12]. IL6 is a pleiotropic cytokine that presents both pro- and anti-inflammatory properties as well as pro- and anti-tumorigenic abilities, depending on the cell type and conditions. It is also involved in metabolism control [76]. Its overexpression in cancer has been linked to cachexia [77], a drawback in the therapeutic use of ONC. Nevertheless, this effect may be partly counterbalanced by the ONC-induced overexpression of myocyte enhancer factor 2C (MEF2C), which is found among the 20 most ONC up-regulated genes (Table 1). MEF2C is a myogenic transcription factor that plays a critical role in skeletal muscle development and differentiation, but is down-regulated in cachexia [78]. In addition, ONC overexpression of inteleukin-33 (IL33), together with amphiregulin (AREG) (Table 1), may offset the pro-inflammatory properties of IL6 because it has been recently demonstrated that both participate in a pathway of innate immune cell-mediated tissue protection in the intestinal track [79]. Finally, ATF3 has been described as being able to epigenetically regulate the EGR1 expression, leading to attenuation of pro-inflammatory chemokines in human enterocytes [80].

### The mechanism described for ONC cytotoxicity is cell-line independent

Six of the seven ONC differentially expressed genes found in the OVCAR-8 cell line are also differentially expressed in the NCI/ADR-RES cell line (see the Results section). Additionally, in both cell lines, ATF3 and TXNIP are the most ONC up-regulated genes (see the Results section). Furthermore, in OVCAR-8 cell line a slight but significant increase in the expression of the rest of the networked transcription regulators differentially expressed in NCI/ADR-RES is observed, with fold change values between 1.8 and 1.5. These results indicate that the effect induced by ONC on both cell lines is similar. It is also noteworthy that ONC treatment of different hMM cell lines increased the expression of ATF3, GADD45A, and IL6, and involved the MAPK and Jak-STAT signaling pathways [12], suggesting that the cytotoxic mechanism described here for ONC is not cell-type specific.

### ONC causes different pleiotropic effects than those observed for PE5

ONC and PE5, as RNA-damaging drugs, have pleiotropic effects. Focusing on the NCI/ADR-RES results, the pleiotropic effects caused by these RNases are clearly different. ONC affects a lower number of genes (11.5-fold less) than PE5 [28]. Among them, the percentage of up- and down-regulated genes differs between the two RNase treatments. Of the ONC differentially expressed genes, 89% are up-regulated and only 11% are down-regulated, while about half of the PE5 differentially expressed genes are up-regulated and half are down-regulated [28]. This suggests that the ONC initial action is the activation of gene expression, in agreement with the results previously obtained for ONC-treated hMM cell lines [12]. Interestingly, only the expression of 10 genes was altered by both RNase treatments (see the Results section). Among those genes, asparagine synthetase (ASNS) and the phosphoglycerate dehydrogenase (PHGDH) coding genes are worth mentioning. Both are down-regulated by both RNases and are involved in amino acid metabolism, although we have not found any term related to amino acid metabolism either in gene ontology analysis or in the KEGG pathway annotation. PE5 down-regulates more genes related to amino acid biosynthesis than the two mentioned above. Its effect is actually to down-regulate this *de novo* biosynthesis that is significantly increased in cancer cells [28]. In addition, the most PE5 up-regulated gene, Homeobox containing 1 (HMBOX1), is also found among the 20 most ONC up-regulated genes. However, its function is not well known and is controversial [81]. Very likely, these coincidences are only the result of the pleiotropic effects of both RNases. Moreover, among the affected gene ontology terms, only five were common between ONC and PE5, and when compared the KEGG pathway annotation, no terms showed coincidence ([28] and Table 3).

The mechanism used by PE5 to kill cancer cells is primarily related to its ability to down-regulate the expression of genes that code for enzymes involved in the metabolic pathways deregulated in the cancer cells. This decrease in the expression levels of metabolic enzymes is assisted by the down- and up-regulation of several oncogenes and tumor suppressor genes, respectively, that down-regulate the expression of genes coding for these enzymes [28]. The only link between ONC regulated genes and cell metabolism is through the glucose uptake inhibitory effect of TXNIP and ARRDC4 [82, 83].

Taken together, our results indicate that the cytotoxic mechanisms exerted by ONC and PE5 on tumor cells are different, which is consistent with their different cellular localization and perhaps with their different RNA targets, cytosolic RNAs, and nuclear RNAs, respectively. Thus, it is tempting to anticipate a synergistic effect between RNases that exert their action in the cell cytosol and those that do it in the cell nucleus.

### ONC as antiviral agent beyond its nucleic acids degradation ability

ONC has been described as having antiviral activity [25]. Due to the ability of wild type ONC and several ONC variants to cleave different types of RNA [10–15], as well as dsRNA [24], their antiviral properties have been mainly linked to degradation of viral genomes and/or host-cell nucleic acids needed for viral replication [25, 84]. However, the finding that ONC cell treatment promotes the up-regulation of ATF3 transcription factor independently of the cell type, leads to the proposal that other mechanisms can strengthen ONC antiviral action. ATF3 is a transcription factor whose role in cancer is clearly cellular-context dependent, but it seems essential for the maintenance of host defense mechanisms [34].

Genomic integration of HPV DNA, occurring in more than 90% of cervical cancers, results in viral E6 protein expression that can interact with a cellular ubiquitin ligase E6-associated protein (E6AP) and target the tumor suppressor p53 for ubiquitin-mediated proteolysis [85]. ATF3 interaction with viral protein E6 prevents p53 from ubiquitination and degradation, leading to expression of p53-target genes, cell cycle arrest, and apoptotic cell death. Thus, ATF3 is a repressor of the oncogenic viral protein E6 and blocks HPV-induced carcinogenesis [86]. Moreover, the ONC overexpressed gene that codes for protein tyrosine phosphatase receptor type R (PTPRR) has been linked to the inhibition of MAPK signaling through dephosphorylation of p44/42 MAPK, which inhibits AP-1 and the subsequent expression of the E6 and E7 HPV oncoproteins responsible for cervical cancer initiation and progression [87].

After infection at a portal entry, herpes simplex viruses (HSV) infect sensory nerve endings and are retrograde transported to the neuronal nucleus, where they establish a silent or latent state. It is not well known how these virulent viruses remain silent. However, it has recently been described that ATF3 plays a key role in the maintenance of the HSV in this latent state by increasing the expression of a non-coding RNA known as the latency-associated transcript (LAT) [88].

Finally, ONC also inhibits 90-99.9% of human immunodeficiency virus type 1 (HIV-1) replication in H9 leukemia cells over a four-day incubation at concentrations not toxic to uninfected H9 cells [25]. HIV-1 transactivator protein Tat uses CREB to promote IL10 production. Although the importance of this for HIV pathogenesis is not clear, IL10 can inhibit HIV-1 replication in monocytes and macrophages, suggesting that Tat/CREB-induced IL10 production provides a negative feedback signal to prevent excess HIV-1 replication [89].

## CONCLUSIONS

Our results explain the cytotoxic mechanism induced by ONC in treated cancer cells through a network of different ONC up-regulated genes. Among them, ATF3 up-regulation plays a central role in the key events triggered by ONC that finally lead to apoptosis. This mechanism is cell-type independent. Up-regulation of ATF3 may also explain the antiviral properties of this RNase. ONC-affected genes are different from those affected by nuclear-directed RNases. The pleiotropic effects of both types of RNases make them attractive as therapeutics to treat either cancer or virus-promoted diseases.

## MATERIALS AND METHODS

### ONC expression and purification

Construction of plasmid expressing ONC (pONC) has been previously described [90]. Recombinant ONC was produced and purified from Escherichia coli BL21(DE3) cells transformed with pONC essentially as described in [91]. The molecular mass of ONC was confirmed by matrix-assisted laser desorption/ionization time-of-flight (MALDI-TOF) mass spectrometry at the Unitat cientificotècnica de suport, Institut de Recerca, Hospital Universitari Vall d’Hebron (Barcelona, Spain). ONC concentration was determined by ultraviolet spectroscopy using an extinction coefficient at 280 nm of 10470 M^-1^ cm^-1^, calculated as reported [92].

### Cell lines and culture conditions

NCI/ADR-RES human ovarian cancer MDR cell line (formerly MCF-7/ADR) [93] was a generous gift from Dr. Ramon Colomer of the *Institut Català d’Oncologia de Girona, Hospital Universitari de Girona Dr. Josep Trueta* (Girona, Spain). It was initially obtained from the American Type Culture Collection (ATCC) (Manassas, Virginia) and was used immediately after thawing. The OVCAR-8 human ovarian cancer cell line was obtained from the National Cancer Institute's DCTD tumor repository (Frederick, USA) and was used immediately after thawing. NCI/ADR-RES and OVCAR-8 cells were routinely grown at 37°C in a humidified atmosphere of 5% CO_2_ in DMEM (Gibco, Germany) containing 1.84 μM doxorubicin (Tedec-Meiji Farma, Spain) and RPMI (Gibco, Germany), respectively, supplemented with 10% fetal bovine serum (Gibco, Germany), 50 U/ml penicillin, and 50 μg/ml streptomycin (Gibco, Germany). Cells remained free of *Mycoplasma* and were propagated according to established protocols.

### Cell proliferation assay

NCI/ADR-RES cells (10^4^ per well) and OVCAR-8 cells (1.5 × 10^3^ per well) were seeded into 96-well plates. After 24 h of incubation, cells were treated for 24, 36, or 48 h with various concentrations of ONC, ranging from 0.001 to 10 μM. Sensitivity to RNase was determined by the 3-(4,5-dimethylthiazol-2-yl)-2,5-diphenyltetrazolium bromide (MTT) method used according to the manufacturer's instructions (Sigma, USA). Data were collected by measuring the absorbance at 570 nm with a Synergy 4 multi-well plate reader (Biotek Instruments, USA). The IC_5_, IC_10_, and IC_15_ values represent the concentrations of ONC required to inhibit cell proliferation by 5, 10, and 15%, respectively, and were calculated by interpolation of the obtained growth curves. Data were calculated as the mean ± SD of three independent experiments conducted in triplicates.

### ONC treatment and RNA isolation

NCI/ADR-RES cells (2 × 10^5^ per well) and OVCAR-8 cells (5 × 10^4^ per well) were seeded into 6-well plates. After 24 h of incubation, cells were treated for 36 h with a concentration of ONC that caused a 10% decrease in cell proliferation (0.5 μM ONC for NCI/ADR-RES cells and 0.06 μM ONC for OVCAR-8 cells). Cells were then harvested at 400xg for 5 min at 4°C and washed twice with cold PBS. Total RNA was extracted using the mirVana miRNA isolation kit (Applied Biosystems/Ambion, USA) according to the manufacturer's instructions and stored at -80°C. Four independent preparations were performed for each cell line. The 260/280 nm absorbance ratio of each sample was checked using a NanoDrop ND-1000 spectrophotometer (Thermo Fisher Scientific, USA). RIN values were used to check RNA integrity using an Agilent 2100 bioanalyzer (Agilent Technologies, USA). The RIN value corresponds to the ratio of ribosomal band areas to total area of the electropherogram and the height of the 18S peak [94].

### Gene expression microarray analysis

Gene expression microarray experiments were performed at Bioarray, S.L. (Alacant, Spain) using the SurePrint G3 Human Gene Expression Microarray (Agilent Technologies, USA), a high-density oligonucleotide microarray that contains 60,000 probes, corresponding to 27,958 Entrez Gene RNAs and 7,419 lncRNAs. Sample preparation and microarray processing procedures were done according to the Two-Color Microarray-Based Gene Expression Analysis v. 6.5 (Agilent Technologies, USA). Briefly, 200 ng of total RNA were used to synthesize double-stranded cDNA with AffinityScript-Reverse Transcriptase and Oligo dT-Promoter Primer. cDNA was simultaneously amplified and transcribed into cyanine 3- or cyanine 5-labeled cRNA employing T7 RNA polymerase in the presence of cyanine 3-CTP or cyanine 5-CTP. The labeled cRNA (antisense) was purified, evaluated using a NanoDrop ND-1000 spectrophotometer (Thermo Fisher Scientific, USA) and hybridized to the oligonucleotide microarrays at 65°C for 17 h. Microarrays were then washed and scanned on a G2565CA microarray scanner upgraded to a resolution of 2 micron (Agilent Technologies, USA). Data were extracted from the resulting TIFF-images using the Feature Extraction software v. 10.7 (Agilent Technologies, USA). Raw microarray data were statistically analyzed using the Marray, pcaMethods, Limma, and RankProd software packages from Bioconductor (www.bioconductor.org), which uses the R statistical environment and programming language. In particular, the non-specific signal was removed from the total intensity using the normexp background correction method with an offset of 20 [95]. Then intra-slide normalization was done using the Loess method [96] to make intensities consistent within each array, and inter-slide normalization was performed employing the quantiles method [97] to achieve consistency between arrays. After each of these analyses, a quality control analysis of microarray data (RG density plot, MA plot, and M boxplot) was performed. Following normalization, the RankProd method [98] was applied to identify differentially expressed genes. Genes were considered differentially expressed when they had a false discovery rate adjusted p-value ≤ 0.05 and a fold change ≥ 2 or ≤ -2. Data have been deposited in NCBI's Gene Expression Omnibus repository [99] (http://www.ncbi.nih.gov/geo) and are available under the accession number: GSE75494.

### Gene ontology analysis and KEGG pathway annotation

In the NCI/ADR-RES cell line, differentially expressed genes were characterized functionally with a hypergeometric test to find over-represented gene ontology terms in the three main broad ontologies (biological process, molecular function, and cellular component) (www.geneontology.org), and were also mapped to the Kyoto Encyclopedia of Genes and Genomes (KEGG) (www.kegg.jp), which assigns proteins to pathways, to find over-represented pathways. The analyses were done using the GOstats and RamiGO software packages from Bioconductor (www.bioconductor.org). A p-value cutoff of 0.05 was used. The network representation of linkages between ONC-differentially expressed genes was constructed and drawn using the Ingenuity Pathway Analysis (IPA) package available on-line at http://www.ingenuity.com/products/ipa

### Quantitative reverse transcription PCR (*RT*-*qPCR*)

mRNA expression of three ONC up-regulated genes (TXNIP, ATF3, and GADD45A) and two down-regulated genes (PHGDH and ASNS) were examined by RT-qPCR. The same RNA samples used for microarrays analysis were used in this analysis. First, RNA samples were digested with DNase to prevent genomic contamination using the RNase-Free DNase Set (Qiagen, Germany) according to the manufacturer's instructions. They were evaluated using an Agilent 2100 bioanalyzer (Agilent Technologies, USA) and a NanoDrop ND-1000 spectrophotometer (Thermo Fisher Scientific, USA). Then, for each sample, 0.5 μg of RNA was used to synthesize single-stranded cDNA with the High-Capacity cDNA Reverse Transcription Kit (Applied Biosystems, USA) following the manufacturer's instructions. Gene-specific forward and reverse primers for the selected genes were designed with Primer3 (http://primer3.ut.ee) and checked with NetPrimer (http://www.premierbiosoft.com/netprimer/). Primer sequences for ONC-regulated genes were: TXNIP (F: GCTTGCGGAGTGGCTAAAGT; R: CTCACCTGTTGGCTGGTCTTC), ATF3 (F: AGTGAGT GCTTCTGCCATCGT; R: TGACAAAGGGCGTCAGG TTAG), GADD45A (F: GAGAGCAGAAGACC GAAAGGA; R: CAGGCACAACACCACGTTATC), PHGDH (F: TATTGTTCGCTCTGCCACCA; R: TCATAACCAAGATGCCCTTCC) and ASNS (F: AAAGCCGAGGAGGAGAGTGA, R: GGTGGCAGAG ACAAGTAATAGGA). To select a constitutive gene as a reference for normalizing data, the transcription abundances of five genes (ACTB, GUSB, TBP, HPRT1, and ALAS1) were measured for all cDNA samples. Primer sequences are described in reference [28]. Among them, TBP showed the highest stability (lower standard deviation of the Ct; results not shown) and it was therefore selected for data normalization. Real-time PCRs were performed in an optical 96-well plate with an ABI PRISM 7300 Sequence Detector System (Applied Biosystems, USA), using SYBR Green to monitor dscDNA synthesis. Reactions contained 1x Power SYBR Green PCR Master Mix (Applied Biosystems, USA), 300 nM of gene-specific forward primer, 300 nM of gene-specific reverse primer, and 5 μl of a 50-fold dilution of the previously synthesized cDNA in a final volume of 20 μl. The following standard thermal profile was used for all real-time PCRs: 95°C for 10 min, 40 cycles of 95°C for 15 s, and 60°C for 1 min. A dissociation step was performed after amplification to confirm the presence of a single amplicon. To estimate variation in the technique, three technical replicates were carried out for each cDNA sample. Data were analyzed with the 7300 SDS 1.3.1 software (Applied Biosystems, USA). To generate a baseline-subtracted plot of the logarithmic increase in fluorescence signal (ΔRn) versus cycle number, baseline data were collected between cycles 3 and 15. All amplification plots were analyzed with an Rn threshold of 0.2 to obtain threshold cycle (Ct) values. The amplification efficiency for each gene was calculated based on five dilutions of cDNA ranging from 1 to 3.2×10^-4^ and the equation E = 10^(−1/slope)^. All genes had an efficiency value between 1.85 and 2.05. The relative transcription abundances (RTA) of ONC-target genes (TXNIP, ATF3, GADD45A, PHGDH, and ASNS) were calculated as RTA = E^ΔCt(control-sample)^_(Target)_ / E^ΔCt(control-sample)^_(Reference)_ [100], where control refers to a mix of equal amounts of untreated samples. Fold changes were calculated as the ratio between RTA values obtained for ONC-treated cells and those obtained for untreated cells. The absence of genomic DNA contamination was checked using non-retrotranscriptase controls and the absence of environmental contamination using non-template controls.

## SUPPLEMENTARY MATERIALS FIGURES


