# Supplementary material for: Activating transcription factor 3 is crucial for antitumor activity and to strengthen the antiviral properties of Onconase
Source: Oncotarget. 2016 Dec 27;8(7):11692–707. doi: 10.18632/oncotarget.14302 (PMC5355296; doi:10.18632/oncotarget.14302)
Supplement: Supplementary file 1 [file oncotarget-08-11692-s001.pdf]

Activating transcription factor 3 is crucial for antitumor activity and to strengthen the antiviral properties of onconase

SUPPLEMENTARY FIGURES

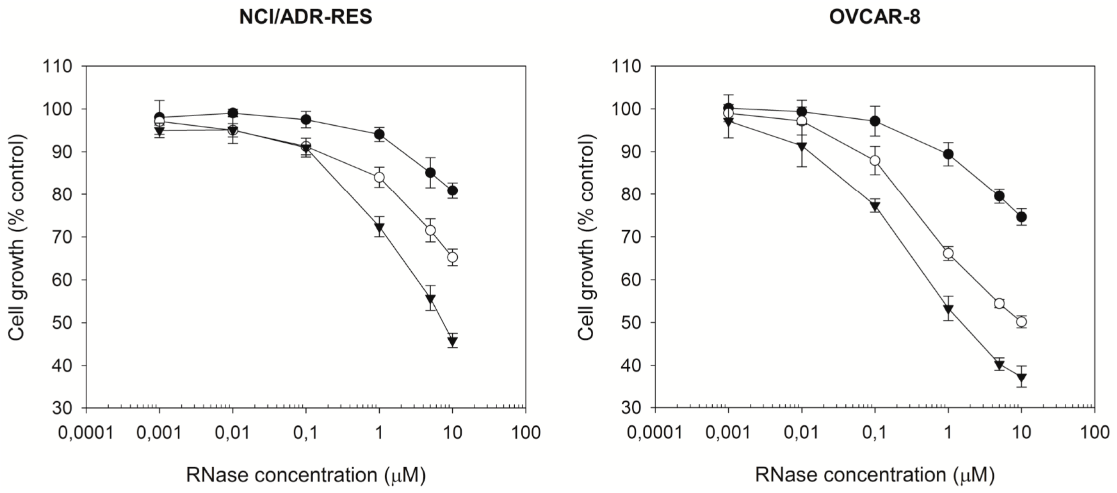

Supplementary Figure 1: Cytotoxic effects of ONC in NCI/ADR-RES and OVCAR-8 cell lines. Control and ONC-treated cells were maintained for 24 (●), 36 (○), or 48 h (▼) and metabolic activity was determined by the MTT assay. Cell growth is expressed as the percentage of activity respective to control cells using the absorbance values. The curves in the figure are from one representative experiment made in triplicates. Data are presented as mean ± SD. Equivalent results were found in at least three independent experiments.

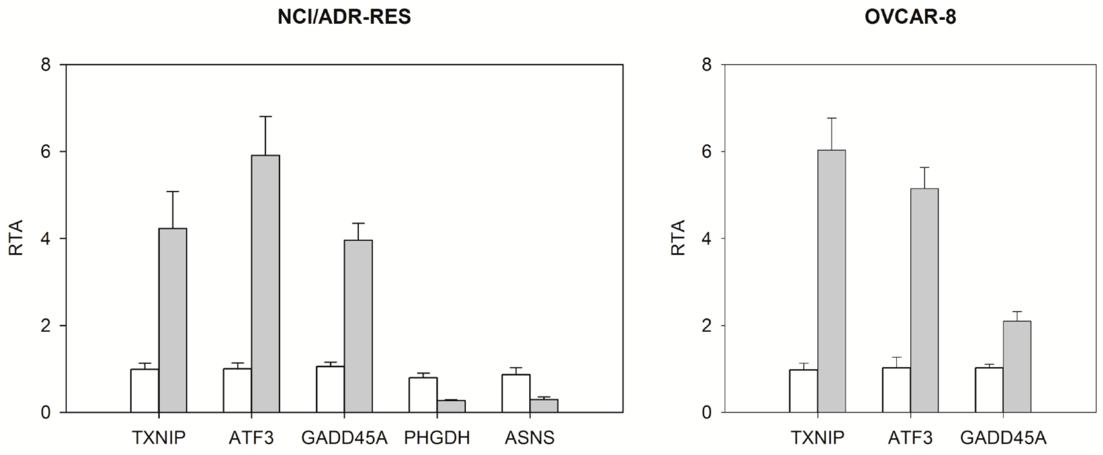

Supplementary Figure 2: Quantitative gene expression changes in NCI/ADR-RES and OVCAR-8 cell lines. The histograms show the relative transcript abundance (RTA) obtained by RT-qPCR of selected genes up- or down-regulated by ONC. White bars, untreated cells; grey bars, ONC treated cells. Data are presented as mean ± SD. Genes: Thioredoxin interacting protein (TXNIP), activating transcription factor 3 (ATF3), growth arrest and DNA-damage-inducible, alpha (GADD45A), phosphoglycerate dehydrogenase (PHGDH), and asparagine synthetase (glutamine-hydrolyzing) (ASNS).
